# Extending Adjuvant Endocrine Therapy for 10 Years: A Mixed-Methods Analysis of Women’s Decision Making in an Online Breast Cancer Forum

**DOI:** 10.3390/healthcare9060688

**Published:** 2021-06-07

**Authors:** Yolanda Eraso, Denes Stefler, Zoe Moon, Leda Rossi, Sidona Assefa

**Affiliations:** 1 School of Social Professions, London Metropolitan University, London N7 8DB, UK; sta0381@my.londonmet.ac.uk; 2 Department of Epidemiology and Public Health, University College London, London WC1E 7HB, UK; denes.stefler@ucl.ac.uk; 3 Health Psychology Section, Institute of Psychiatry, Psychology & Neuroscience, King’s College London, London SE1 9RT, UK; zoe.moon@kcl.ac.uk; 4 College of Nursing, Midwifery and Healthcare, University of West London, London W5 5RF, UK; 21416151@student.uwl.ac.uk

**Keywords:** breast cancer, 10 years, extended endocrine therapy, mixed methods, online forum, women’s decision making, persistence

## Abstract

An additional 5 years of treatment with adjuvant hormonal therapy, to complete 10 years of medication, is recommended to reduce the risk of breast cancer recurrence. Yet professionals and patients should balance this benefit against side effects and toxicities. Little is known about women’s decision making regarding persistence with extended endocrine therapy. In this study, we collected data from a UK online breast cancer forum to analyse patterns of persistence and its associated factors. A mixed-methods exploratory sequential design was used, with a qualitative analysis of text (*n* = 61 individuals) informing the development of a quantitative instrument to statistically analyse the prevalence of the findings (*n* = 130). Our findings identified three different groups of women who had to make decisions regarding persistence with treatment: those about to complete 5 years of therapy, those who decided to extend treatment, and those who were initially prescribed 10 years. Factors affecting persistence were, lack of self-efficacy in managing side effects, lack of reassurance about individual risk of recurrence, and impact on quality of life. Interventions such as training of healthcare professionals including risk communication, medication reviews by clinical pharmacists, and re-planning of services in follow-up care, should better support women’s needs in extended hormonal therapy.

## 1. Introduction

Five years of adjuvant endocrine therapy (AET) for oestrogen-positive (ER+) breast cancer patients has been the gold standard treatment since consensus was reached around the year 2000. For women with early breast cancer, the drug Tamoxifen (TAM) for premenopausal and the drugs Aromatase Inhibitors (AIs) for postmenopausal women have proven to be effective in reducing the risk of recurrence by about half and mortality by about 30% (TAM), and recurrence by about two-thirds and mortality by around 40% (AIs) during the first 15–10 years respectively, after initiation of treatment [1,2].

Despite the benefits associated with 5 years of treatment, early-stage, ER+ breast cancer has a risk of late recurrence and death [3]. Published in December 2012, the ATLAS international trial [4] showed a benefit of extending AET therapy with TAM for 10 years in comparison to stopping treatment at 5 years in women with ER+ and early breast cancer diagnosis. For those who extended treatment, a reduced risk of cancer recurrence (21.4% versus 25.1% in those who stopped at year 5) was observed and a reduction in absolute breast cancer mortality of 2.8% [4]. In the case of AIs, usually prescribed to postmenopausal women, the MR.17R trial [5] found that in women treated with 10 years of letrozole, the risk of disease recurrence was 3–4% lower than women who stopped at 5 years.

Overall, there is a modest (2–5%) absolute reduction in risk of recurrence from extending AET for 10 years [6], yet international guidelines have embraced the extended therapy regime. For many women, however, taking AET medication causes unpleasant and, in some cases, adverse side effects: common side effects to taking TAM are hot flushes, depression, weight gain, and low libido, and less common but severe conditions such as increased risk of pulmonary embolism and endometrial cancer, whereas AIs can cause joint pain, hot flushes, and an increased risk of fractures and osteoporosis [7]. Adherence to the previous 5-year treatment regime was suboptimal [8], and little is currently known about adherence to 10 years of AET. A systematic review [8] found no consistent predictors of persistence with AET, yet some evidence indicated that persistence was associated with receiving treatment by an oncologist instead of a GP, beliefs about AET, social support, and self-efficacy for taking medication. The same study found that in a 5-year AET regime, discontinuation increases from 21% in the first year to 48% in the fifth year [8].

For 10-year extended treatment, data on women’s adherence and persistence is lacking, and so far, there are no studies identifying specific factors associated with these behaviours. One of the challenges for decision making regarding persistence with AET medication is that, unlike other cancer therapies where the regime is much shorter and decision making is taken at a specific point in time (usually at diagnosis), AET decision making is not a one-off event and decisions can change over the course of treatment [9]. In the case of extended therapy, women need to decide if the benefit of extending treatment outweighs the additional side effects that adversely affect their quality of life.

In the UK, guidelines for extended therapy take the burden of side effects into consideration and recommend discussion of the risks and benefits with women, especially those at lower risk of recurrence [10]. However, individualised approaches to identify which women would benefit most is challenging. Although there are various predictive biomarkers (gene-expression assays) to assess benefits from extending therapy, their use is not yet standardised in clinical practice [11]. Instead, clinical factors are used to inform prognostic tools, such as the NHS Predict tool validated in 2011 [12]. Clinical factors include age, tumour size and grade, lymph node involvement, and markers such as the ER, HER2, and KI67 [13]. Taken at the time of diagnosis, and for early invasive cancers only, it can estimate average survival rates associated with different treatment options. In 2018, the ‘clinical treatment score at 5 years’ (CTS5) [14] was introduced, which can predict risk of late recurrence after completing 5 years of treatment. Notably, this tool could help women decide if extending AET was recommended or not, based on the level of risk of recurrence (low, intermediate, or high) [14], although it can overestimate the risk of recurrence in high-risk patients [15].

Arguably, the clinical utility of tests to assess risk is only part of the picture. Patients’ ability to interpret risk and statistics, test accessibility, and the specialist’s role in communicating risk to patients can complicate decision making in women. In addition, there is a range of demographic, psychological, and environmental factors that can affect medication taking in long-term conditions. A recent qualitative evidence synthesis has shown that women in a 5-year AET regime have sought information about their medications in online forums often due to a lack of healthcare support after being discharged from hospitals [16].

Online health discussion forums have grown considerably in the past two decades, and social scientists as well as medical researchers have turned to this type of data to explore different health-related phenomena. In relation to breast cancer, scholars have shown a range of characteristics in terms of online fora’s role for cancer patients (peer support, a safe space to discuss concerns, an empathetic audience to express emotions, a reliable resource to seek for or exchange information and tips) [17,18,19]. In addition, studies have analysed patterns of communication, effects on emotions [20,21], and barriers to treatment for different ethnic groups [22], amongst other topics. Decision making has also been explored in relation to adherence and discontinuation to AET [23,24] and about the use of the Oncotype DX test [25]. Women prescribed a 10-year course of therapy would probably elicit a similar pattern of information-seeking behaviour, but we do not know for certain what type of information women are exchanging and which factors may influence their decisions for continuation or discontinuation. In-depth exploration of these factors may suggest ways to support women with this increased treatment duration and identify ways to help with the decision-making process.

In this paper, we addressed this gap in knowledge by exploring women’s decision making about persistence with AET for 10 years among users of a UK online forum for people with breast cancer. We report on findings from a mixed-methods study where women’s decision to persist with AET medication can happen at different times in their course of treatment and be affected by different concerns. We also identified specific factors that can influence women’s decisions to continue (fear of cancer recurrence, trusting the drug’s effectiveness, and using various coping mechanisms to alleviate side effects), discontinue, and being undecided (poor quality of life, lack of reassurance about individual risk of recurrence, and lack of self-efficacy in managing side effects).

## 2. Materials and Methods

An exploratory sequential mixed-methods research design was developed using the online forum ‘Hormone Therapy’ within the Breast Cancer Now’s Forum in the UK. The study consisted of two phases: Phase (I) used a qualitative approach, and the findings informed the development of a quantitative instrument (Phase II) to statistically analyse the prevalence of the findings in a larger sample of women.

### 2.1. Study Setting

The Breast Cancer Now’s Forum [26] is the largest of its kind in the UK, and unlike other cancer forums, its content on treatments is clearly organised around different boards: radiotherapy, surgery, chemotherapy, targeted therapies, and hormone therapy. The forum’s content is accessible to anyone (16 years or older) on the Internet, but to post, individuals need to register. Forum guidelines contain recommendations to users regarding privacy and safety (i.e., no disclosure of personal details) as everything posted is accessible to the public. Moderators ensure that forum guidelines are complied with. A dedicated group of breast cancer nurses offer specialist support to users, and a group of community champions, who are women with breast cancer, provide support and empathy to other users’ posts. Most forum users are women undergoing treatment, although men with breast cancer, family members, and women who have completed or discontinued treatment can also post. As a UK-based charity, the great majority of users are from the UK, but there is no geographical identification of users. In our analysis, we found posts from women outside the UK, in particular from English-speaking countries, who have explicitly mentioned the countries where they currently live. Overall, the forum provides a great variety of experiences as women from different socio-demographic characteristics and places can join the discussions. At the time of starting data collection, the forum had 56,419 registered members [26].

### 2.2. Qualitative Phase (I)

Data was collected from the ‘Hormone Therapy’ online forum by two research assistants (S.A. and L.R.) with experience in qualitative research and supervised by Y.E., who is an experienced qualitative researcher with previous studies on adherence to hormonal treatment in breast cancer patients. At the time of data collection (February–March 2019), the ‘Hormonal Therapy’ forum contained 181 threads. We used a purposive sample to select posts within the forum. We included women prescribed 10 years of hormonal treatment, for any stage of breast cancer, for the drugs TAM or AIs, either pre-menopausal or postmenopausal, expressing decision making regarding persistence, between the period 1 January 2013 and 31 January 2019. We excluded from our sample: men with breast cancer and family members, not in a 10-year treatment, and not expressing decision making.

We randomly selected 70 threads, which contained a total of 1054 posts. After applying the inclusion/exclusion criteria, 24 threads were relevant, and 79 posts were included in the analysis, which were contributed by 61 women. All posts from the threads were read and relevant posts were subsequently copied and pasted on a Word doc. recording thread title, number of posts contained within the thread, and posts included.

Thematic analysis was applied to the data, following the six steps outlined by Braun and Clarke [27]. Y.E., S.A., and L.R. read and re-read the posts, and each generated analytical notes that were then discussed in meetings. Thirty-two posts were independently coded line-by-line by Y.E., S.A., and L.R. using an inductive (women’s narratives) and deductive approach (literature on persistence to AET medication). Codes were iteratively revised in team meetings. Y.E. and S.A. then applied the codes independently to the remining data, before a discussion and final revision of codes was performed. Codes were then grouped together into themes and sub-themes and were discussed in team meetings. Theme identification first captured different points at which women made decisions about persistence with medication. Patterns that characterised each of these themes and theme names continued to be refined by Z.M. (a health psychologist with research expertise on adherence to AET medication) and Y.E. until the writing stage of this article.

### 2.3. Quantitative Phase (II)

Data were collected by L.R. and S.A. for the 181 threads available in the ‘Hormone Therapy’ forum, following the list of codes identified in the qualitative analysis. An Excel matrix was used with one column for each code, one column for each treatment scheme group (planning, in 10-year treatment, and initially prescribed 10 years), one column for each decision made (continuation, discontinuation, and undecided), and one individual per row. Extracted data were cross-checked by a second reviewer (Y.E.). Data were subsequently collected by Y.E. for the 130 women identified for two demographic variables: (1) women’s age and (2) living with family or alone.

Information on treatment scheme, treatment duration, and the decision regarding treatment continuation was available for all included participants, while data on their age and family status could be extracted for 76 and 66 individuals, respectively.

In the first step of the analysis, the proportion of participants whose post comments indicated that they (1) continued or (2) discontinued with the hormonal treatment, or (3) still undecided was calculated across categories of treatment scheme, treatment duration, age, and family status. Statistical significance of the respective differences was assessed using Chi-square tests.

In the second step, the codes identified in our qualitative data as factors influencing decision making were considered. The proportion of individuals who mentioned the specific codes was calculated in the overall sample, and also by categories of participant characteristics, such as treatment scheme, decision regarding treatment continuation, treatment duration, age group, and family status. The statistical significance of the differences of proportions across categories was estimated using Chi-square tests.

Finally, the associations between selected codes mentioned in the posts and the decision regarding treatment continuation were assessed using crude (unadjusted) and multivariable adjusted logistic regression models. In the multivariable adjusted models, the associations were adjusted for treatment scheme, treatment duration, age group, and family status. Individuals with missing data on age and family status were grouped in a separate category for each, therefore the analysis was run on the full sample size.

**Figure 1:**
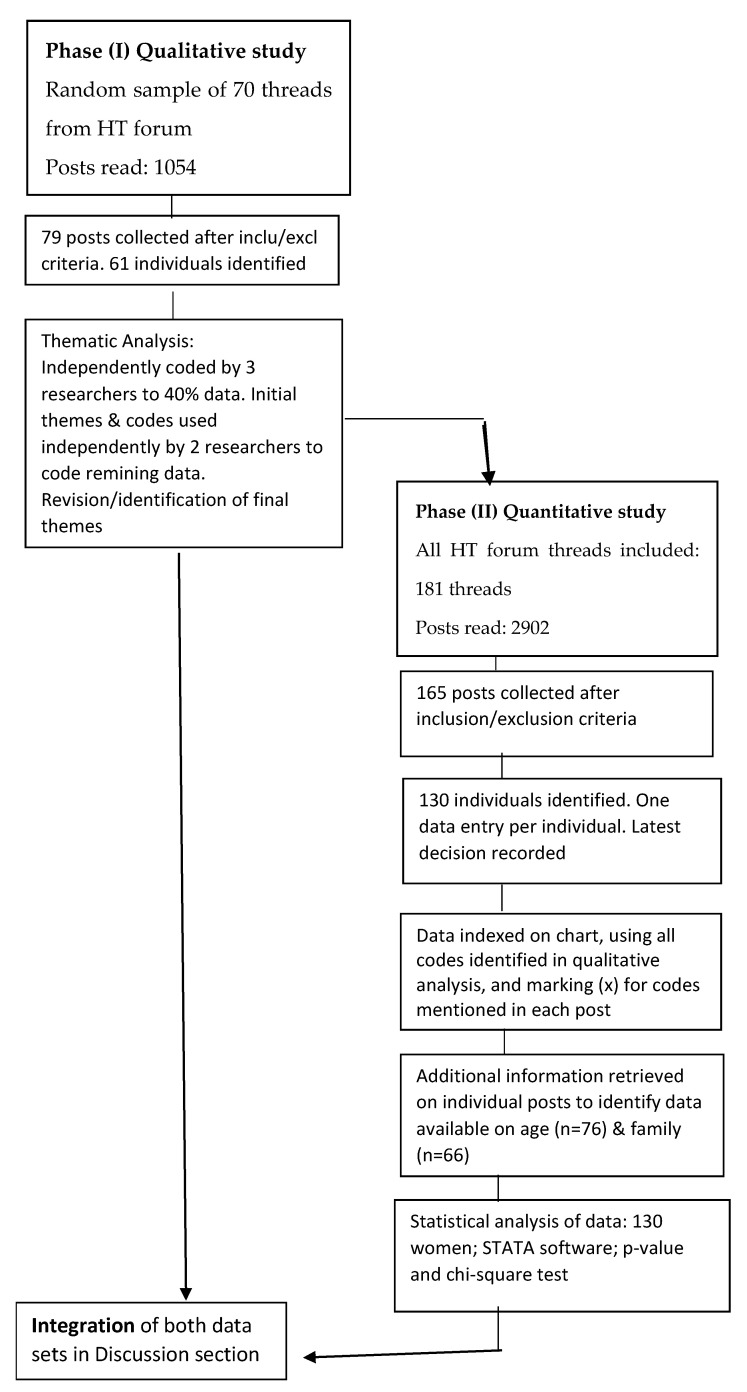
Flowchart of the mixed-methods design.

All statistical analyses were carried out using the software STATA v15.1 (StataCorp, TX, USA). Figure 1 illustrates the steps taken in the two phases of the study.

### 2.4. Data Integration

Data integration is presented in the Discussion Section. In bringing the data together, we aimed for ‘expansion’ as quantitative data had the purpose of explaining the strength of the associations observed in the qualitative data [28].

## 3. Results

### 3.1. Qualitative Analysis

Narrative posts from 61 women were included in the analysis. Five themes were identified in the data: Disappointment, key drivers of decision making (subthemes: fear of recurrence/necessity beliefs, side effects/QOL, and impact of family), lack of support or information from the healthcare team, utilising risk information, and coping mechanisms.

In addition, we identified three groups according to women’s treatment scheme: (G1) Planning 10-year treatment (currently in 5 years), (G2) in 10-year treatment (originally in 5 years), and (G3) prescribed for 10-year treatment. Groups are indicated in the extracts, alongside the years in treatment and the decision made (continue, discontinue, or undecided).

Extracts presented from women were de-identified by using pseudonyms and by paraphrasing the original text posted online, as required by the Ethics committee that approved this study.

#### 3.1.1. Disappointment

For women who had been initially prescribed 5 years of AET, the notion of continuing treatment beyond 5 years came as a disappointment, even for those who decided to continue. Most women felt that they had put up with side effects for the 5 years and had come close to the milestone, when they were suddenly asked to continue.

I have taken Arimidex for two and a half years. When I consulted my doctor about all the awful side effects, and what would happen if I stopped taking it, he replied that the protocol had changed and now it is for 10 years!! I started crying…I was devastated by the prospect of “living” that way.

(Helen, G1, 3+ years, discontinued)

I was resigned to the side effects as I expected to be done after 5 years, only to be told current research and statistics indicate 10 years to be better than 5.

(Myriam, G2, 5+ years, continued)

#### 3.1.2. Key Drivers of Decision Making

##### Fear of Recurrence/Necessity Beliefs

Many women were not happy about needing to continue for a further 5 years of treatment but were motivated by the need to reduce the risk of a recurrence.

When I was about to complete my 5 years, I had mixed feelings. On the one hand, it would have been great to give up all medication and return to my former self (hopefully). On the other, I just wasn’t prepared to take the chance… No, I’m not happy taking the drug, but it beats the alternative.

(Andrea, G1, 5 years, continued)

One woman reported coming off Letrozole in her 9th year of treatment but being driven to restart the medication due to fear that the cancer might come back.

I did a full body scan and became worried that the cancer may have come back. I decided that there was no point in being worried and so I’m now back on the drug until I am due to finish next year.

(Adele, G3, 9 years, continued)

Some women had discontinued treatment due to side effects, and for some, this increased risk of recurrence was reported as difficult to deal with. There was some uncertainty as to how to know if the right decision had been made.

I’m now living in fear of recurrence. I’ve been off tamoxifen for months now. I sympathise with you all.

(Linda, G2, 7 years, discontinued)

I am worried as my oncologist wants me on the medication for 10 years, but there’s no way... I just hope my decision is the right one.

(Laura, G3, 4 years, discontinued)

##### Side Effects/QOL

For many women, the decision to not continue for the full 10 years was driven by a desire to avoid side effects and maintain quality of life.

I just been informed that the guidelines have changed, and that I will need to continue with the medication for a total of 10 years! I said ‘NO’. I used to be very active and fit when I started treatment at 52 years old. Now, after 4 years, I’m unable to do any of the activities I used to do and enjoy! I am 56 but feeling 96! I work FT, but by midday, I’m really fading and struggling to concentrate. The challenging thing is: we have life (and I’ve lost dear friends through breast cancer) but without the quality, leaving a yoyo of emotions. Inside, I’m jumping for joy, but the Letrozole prevents me from doing the physical jumping! Good luck everyone.

(Elizabeth, G1, 3 years, discontinued)

Many women focused on their age and described how the medications made them feel older and prohibited them from doing the things that they wanted to do. This was also a concern for women who were motivated to stay on the medications but were weighing these up against the cost of the side effects.

I’ve been on Tamoxifen for 7 years and I decided to come off. I’m 52, but felt like an old woman. I even struggled to get out of the bath. So I discussed stopping taking TAM with my consultant and he said yes. […]

(Linda, G2, 7 years, discontinued)

Just about to turn 57 years old, but I’m feeling, thinking and moving like an old lady. I can’t walk properly, and I’m in constant pain. I understand that the Anastrozole is helping to keep me alive, but at what cost? It’s a trade-off, stay alive, but without quality of life, or risk it and enjoy living? The question is ‘What to do’?

(Anne, G3, 8 years, undecided)

Other women suffered from risky medical conditions like endometriosis that led them to discontinue, despite suffering from other side effects.

I have been taking Tamoxifen for 6 years. Now ready to come off!! I have many side effects (hot flashes, pain in my legs and bones, dizziness, blurry vision, but I manage with it). My main concern is the risk of endometrial cancer as my uterine lining continues to grow.

(Jo, G2, 6 years, discontinued)

##### Impact of Family

Consideration of family was also a key decision maker for some women. One woman described her young daughter being her motivation to continue taking a treatment, despite the side effects.

The side effects were a pain… but I told myself… you have lots to lose if you don’t take the drugs. My cancer was the ‘spreading fast’ type, and I had a child of 13 years old then. I am a single mum, and looking into her heartbroken eyes, made me realised I needed to do the tamoxifen, whether I liked it or not. I don’t regret the choice I made, because I’m in remission and it has been almost 10 years.

(Hannah, G3, 9 years, discontinued a few months earlier)

Conversely, being older and without family influenced some women to prioritise their quality of life rather than coping with side effects.

I am 68 and without family. My quality of life is very important. I’ve read about the treatment being for 5 or 10 years. I’d probably live into my 80s, maybe in poor health, with some problem or other.

(Mia, G3, about to start, undecided)

#### 3.1.3. Lack of Support or Information from Healthcare Team

Some women reported a lack of trust with their healthcare professional. They felt that they were not provided enough information about their personal risk of recurrence in order to make a fully informed decision.

Nobody will give me a straight answer regarding my risk of recurrence if I stop Tamoxifen after 5 years.

(Louise, G1, 5 years, undecided)

Others felt confused as to why they had not been told about the 10-year prescription from the beginning of their treatment.

I wonder why, since the oncologists knew about doing 10 years before my diagnosis, which they did, why wait until now? when I am about to complete my treatment. Now they say ‘oh, do more years’.

(Jasmine, G1, 5 years, undecided)

Many women also felt dismissed, or not understood by their healthcare teams when they tried to explain the side effects that they were experiencing. They reported that their doctors did not understand what it is like for them to take the medications and often refused to attribute issues to the hormone therapy.

At one point, I told my oncologist that I felt depressed, and she prescribed me anti-depressants...however, I didn’t take them, I didn’t think she actually understood my needs.

(Karim, G1, 3 years, discontinued)

I have the feeling that doctors don’t really understand what side effects are… They just follow what they learn from studies and protocols.

(Jasmine, G1, 5 years, undecided)

My oncologist didn’t think the tamoxifen was giving me pain in my calf muscles as I’ve been taken it for 4 years. He insisted I would have noticed the pain sooner. He was WRONG. I am still doing physiotherapy to deal with all the damage caused by the drug in my legs. In 2 months since I stopped taking it, the pain has not come back.

(Rosa, G1, 4 years, discontinued)

#### 3.1.4. Utilising Risk Information

Linked to the above theme, some women felt that they were not given the right information necessary to make this decision, and therefore had to do their own research. This largely involved using the Predict tool to determine their personal level of survival with AET. Most women who were told they were low risk (through other tests) used this to justify or drive their decision to discontinue treatment.

I heard about the predict tool, asked my new oncologist why I didn’t have the Ki67 data, and he told me that I didn’t need it as I was low risk for recurrence. I was shocked about doctors not telling me this. He agreed I will stop taking it. … Now I am happy to be called ‘non-compliant’ and hope I had been proactive sooner.

(Karim, G1, 3+ years, discontinued)

However, a few women who had used the Predict tool still found it hard to make a decision and to interpret the information. They were worried about the risk of the cancer coming back, even if they were given non-significant benefits in terms of survival.

Apparently, aromatase inhibitors give me 1% greater survival over a 5-year period and 2% survival if I take it for 10 years. My only concern is I don’t know if this is a very small risk and could stop taking it, or if 1 in 100 is a big risk…I wonder how you make a decision?

(Sofia, G1, 4 years, undecided)

#### 3.1.5. Coping Mechanisms

Some women mentioned switching medications as a way of coping with the side effects and helping them to stay on the medications for longer. This was often initiated by the woman’s healthcare team but led to many women recommending this to others.

My oncologist at some point switched me to Exemestane…I take the brand name and feel so much better. Maybe you could find out and give it a try. All the best.

(Diana, G3, 5+ years, continued)

Similarly, others tried different brands in an attempt to avoid bothersome side effects and continue with treatment, and ultimately, improve their quality of life.

I am doing my best to jot down the name of the brands that works better for me, if there is one, and I am confused.

(Martha, G3, 6 weeks, continued)

For a few months I took a different brand, and suddenly I suffered depression and stronger joint pain, etc., but this went away as soon as I got back to my usual brand.

(Mary, G3, 5 months, continued)

Others described missing doses in order to reduce the intensity of their side effects. This was perceived as a way to retain the benefits of the treatment whilst minimising the intensity of the side effects.

I noticed many women give up Anastrozole because they can’t tolerate the side effects, but I think that is worst, as it increases the risk of recurrence due to oestrogens rising again. It’s better to take one every other day, so at least you don’t give it up completely.

(Sandra, G3, a few months, continued)

Many women resorted to other mechanisms such as changing diet, physical activity, and psychological therapies, amongst others, to help them manage side effects and maintain quality of life. A woman who said she was very tired during the day due to lack of sleep induced by night sweats, stated:

I started acupuncture las week at my hospital’s centre. So far, I haven’t had night sweats and my hot flushes are fewer in the past days. I have to take the medication for 10 years as I am high risk. Coming off the drug is not an option!

(Maribelle, G3, 2 months, continued)

### 3.2. Quantitative Analysis

Table 1 shows the number and proportion of individuals in the three decision-making categories (continue, not continue, undecided) across treatment scheme, treatment duration, age, and family characteristics. We found that more than half (55.6%) of the overall sample had decided not to continue with treatment or were undecided. Those who were currently in 5-year treatment and planning to extend it to 10 years (G1) were significantly more likely to be undecided or have already decided not to continue than those who were already in the 10-year treatment groups (G2 and G3). Women whose treatment had been going for 2–5 years were significantly more likely to be undecided or not to continue with the treatment compared to those with shorter or longer treatment duration. However, this observation is most likely due to the fact that the majority of individuals in the 2–5-year duration category were those who were in the planning stage (G1). Among participants with available data, there was no significant difference in the decision made on treatment continuation according to the women’s family status (i.e., live alone or with family) or age group.

Table 2 shows the proportion of women who mentioned the codes identified in our qualitative data as factors influencing decision making, across the whole sample and within different treatment schemes and decision-making groups. 

The most common topics were side effects of medication (92%), quality of life affected by medication (35%), and not coping with side effects (25%). With regards to different treatment schemes, the only significant difference across groups was found for the expressed lack of trust in relation to the medication and towards health professionals. Those who were currently planning the 10-year treatment were more likely to mention this issue compared to the other two groups.

In terms of the decision about treatment continuation, we found several codes that showed significant differences across the categories. Women who decided to continue with the treatment were more likely to mention fear of cancer recurrence, the fact that they trusted the drug’s effectiveness, and that they used various coping mechanisms in order to alleviate the side effects. On the other hand, participants who decided not to continue with the treatment or were still undecided were significantly more likely to mention the impact of medication on their quality of life and that they did not trust health professionals. These groups also more often indicated that they did not cope with the side effects.

We further explored the frequency of mentioning these codes across age groups, family status, and treatment duration (Table S1 in Supplementary Materials). The large majority showed no statistical differences between the categories of these characteristics. One interesting finding is that researching the disease using reliable sources was more common in those who live alone compared to women who live with family.

Table 3 shows the crude and multivariable adjusted association between selected codes mentioned in the posts and decision on treatment continuation. The results indicated that mentioning the fear of cancer recurrence, trusting the drug’s effectiveness, and using various coping mechanisms to alleviate side effects remained significantly more common in those who continue with the treatment, even when the treatment scheme, treatment duration, age, and family status were taken into account. Similarly, impact on the quality of life, the lack of trust in the medication or health professionals, and the inability to cope with side effects remained statistically significantly more common in undecided participants and in those who discontinued with the treatment compared to individuals who continued, even after multivariable adjustment.

## 4. Discussion

This study offered a novel insight into the decision-making process that women engaged in when asked to extend AET for 5 additional years or initiate a 10-year treatment. The qualitative and quantitative data complement each other, with the former exploring the interplay of factors determining women’s decision making (nature of associations) and the latter revealing the strength of associations amongst the factors identified. Our timeframe for data collection (2013–2019) allowed us to identify three distinct groups (treatment schemes) where decision making on persistence with 10-year treatment offers insights into patterns and associated reasons for continuation, discontinuation, or being undecided. These groups differed by their treatment scheme, as explained below.

G1, planning 10-year treatment (currently in 5 years): Women for whom an extension came as a surprise, at the time or near the time they were about to achieve a milestone, i.e., the 5-year mark. Only around 20% were planning to continue, constituting the lowest percentage of all groups. Fear of cancer recurrence was a key reason to extend treatment, even when women mentioned many bothersome side effects, i.e., necessity/concern framework [29], as observed in studies of AET adherence and medication-taking behaviours [30,31]. In some cases, this decision was also supported by trust in the oncologist. Quality of life was a consistent factor in women’s decision not to continue with the medication. For some patients, the decision to discontinue was arrived at in discussion with the oncologist, after actively seeking research papers and demanding the Predict tool from their professional to assess the added benefit of continuing with AET. Those who were told they had a small risk of cancer recurrence, decided to stop. Other women expressed a lack of trust in healthcare professionals about what they perceived as GPs/oncologists not providing information and downplaying side effects, as observed in other studies [32,33,34]. Another related issue was oncologists and GPs’ lack of reassurances about women’s risk of recurrence, leaving some of them undecided regarding treatment continuation/discontinuation.

G2, in 10-year treatment (originally in 5 years): This group of women had already decided to extend treatment after initially being told they were on a 5-year course of AET. Yet after making a momentous decision, they were confronted again with new bothersome side effects or life-threatening conditions that led them to re-evaluate their continuation. Women complained about newly experienced side effects as well as the worsening of known ones that became evident during the extended period. This led many women who were undecided to seek help from the forum and to ask their oncologists to switch medications, as they felt the side effects from AIs were much harder to tolerate. Interestingly, others tried different brands of the same medication to see if they could tolerate them better. Those that reported continuing with treatment (56%) usually referred to easing of side effects or succeeding with various coping mechanisms: creams, diet, adjunctive medication, psychological therapies, acupuncture, yoga, amongst others, some of which have been recently assessed as evidence-based interventions to alleviate specific symptoms [35]. In addition, fear of cancer recurrence was often mentioned as a reason for continuation.

G3, prescribed for 10-year treatment: Women in this group were prescribed 10-year treatment at diagnosis, of whom around 64% were planning to continue, however, there were 23% undecided. This is unsurprising as women often expressed concern and uncertainty about the prospect of long years of treatment. Age seemed to play a role, but in combination with feelings for family members (e.g., looking after children), as a key issue for women to persist, despite side effects. Conversely, being older and without family influenced women to prioritise their quality of life rather than a perceived long and unpleasant treatment. Although older age (70+) has been found in 5-year studies to be an independent factor for discontinuation with AET [36], for an extended therapy, this may include younger ages (60+) as only 50% decided to continue with treatment in our sample. Trust in medication effectiveness led some women to be persistent whilst becoming non-adherent (skipping doses), indicating misinformation on how the drugs work. In addition, many women proactively asked oncologists to switch their medication, usually after doing research on the Internet or asking the online forum. Often oncologists were able to change their course of treatment from TAM to AI or vice versa, and if tolerant, they continued with the new drug. There is evidence that switching medication within AIs (Letrozole, Anastrozole, and Exemestane) is an effective intervention [35], yet for the association of continuation and switching between TAM and AIs, the evidence is still inconsistent [37].

We believe that our study is the first reported on women’s decision making to take AET for 10 years. This is a striking gap in the literature given that extended therapy has been used since 2013. We have identified factors influencing women’s decisions, which confirm some of the findings observed in previous research focusing on the traditional 5-year treatment: fear of cancer recurrence, side effects, belief in medication (trust/distrust of its effectiveness), information, and support from health professionals at the time of diagnosis and in follow-up appointments. In addition, our findings also revealed the impact of other factors for an extended treatment: self-efficacy in managing side effects, reassurance from healthcare professionals about the risk of recurrence, and new coping mechanisms (changing medication brands) to improve quality of life. Below, we discuss these factors alongside implications for practice.

With regard to side effects, our data indicates that the majority of women reported experiencing bothersome side effects in the overall sample (92.5%), yet the percentage of women who expressed not coping with side effects was considerably lower (25.4%). The latter was also associated with those undecided and discontinuing in the multivariate analysis, which suggest that experiencing side effects alone may not be positively associated with discontinuation, but it needs to be interpreted alongside self-efficacy for managing side effects (i.e., ability to cope). This is supported by research showing that self-efficacy for coping with symptoms can reduce the negative impact of side effects in breast cancer survivors [38]. Interventions to support this include a range of pharmacological and non-pharmacological interventions to control the impact of hot flushes, sexual dysfunction, joint pain, weight management, and fatigue, and have been recently updated according to the best available evidence base [35].

Women reporting a lack of trust in healthcare professionals (9.2%) were often referring to the lack of reassurance on their risk of recurrence, for which some of them requested further prognostic tests (4.6%). Although the percentage of those actively seeking a test was relatively low, many more women in our qualitative data expressed uncertainty about interpreting their risk and sought advice from the forum, something not captured in our quantitative sample. The latter is surprising as both the guidelines [10,39] and specialised literature [5] have consistently emphasised that oncologists should discuss with women the impact of side effects on quality of life alongside the benefits (individual risk of recurrence) of adding 5 more years of therapy. Training oncologists and GPs (in the UK, GPs are involved in follow-up care and use prognostic tools with patients) on how to communicate risk that is accurately understood by patients is of the most relevance to avoid unnecessary stress and decision regret. There are techniques that have been proven to work, such as the development of a patient decision aid combining pictograph, specific numerical information, and text to communicate genetic risk. One study reported more accurate risk estimates regardless of the individual’s numeracy skills [40]. Additionally, of relevance seem to be changing medication brands (7%), as women drew on this strategy to persist with treatment and regain some quality of life. Again, discussion on this topic was more prominent in the qualitative data analysed, with quantitative data only reporting on those who did change it. The NHS Structured Medicine Review, led by a new clinical pharmacist workforce in Primary Care Networks, could provide an opportunity to reassure patients on how different brands for the same drug work, and what side effects are more likely to occur. The SMR could be well-suited to deliver this as they are meant to facilitate “shared decision-making conversations with patients aimed at ensuring that their medication is working well for them” [41] (p. 2).

Finally, the percentage of women ‘undecided’ (28.5%), as shown in our study, deserves more scrutiny as studies on persistence have traditionally identified continuation/discontinuation only. Knowing that a good proportion of women can feel at some point undecided about extended treatment should inform revisions in follow-up guidelines and healthcare professionals’ training. A named contact with a breast cancer nurse should be streamlined as part of the care package, as nurses could provide referrals with oncologists should they need to. Much as online forums provide support in signposting for women about where to seek advice, follow-up care should be adapted to support extended therapy.

### Strengths and Limitations

Online health discussion fora constitute a rich set of data to explore accounts on women’s decision making for extending AET therapy. Due to the observational ‘naturalistic’ nature of this research, the content of the posts is likely to reflect the women’s true feelings and is unaffected by desires to please the researcher or give a socially acceptable answer. However, there are some limitations in dealing with an unwieldy and user-led type of data. Demographic data were not possible to retrieve for all selected users and information provided in posts can be incomplete or sometimes difficult to fully comprehend. To mitigate for the latter, we followed, as much as possible, users’ posts through other threads in the forum to ensure consistency in our interpretation.

Women who post in online forums may not be representative of the general population. Therefore, the results, particularly in terms of the quantitative analysis, may not be entirely generalisable to all women in breast cancer treatment. The moderate sample size is another limitation for the quantitative analysis. However, the fact that we did detect significant associations suggests that such analysis can be meaningful even in a relatively small sample. The calculated statistical power for those key factors which were analysed with the regression method (Table 3) ranged between 0.44 and 0.99. This suggests that our analysis had sufficient power to detect significant differences across treatment continuation groups regarding most of the examined factors mentioned by the participants. Nonetheless, we acknowledge that for many other factors presented in Table 2, the sample size was too small to detect existing differences as statistically significant, and future studies would require higher sample sizes to analyse these factors further.

Some scholars [22] and members of the Breast Cancer Now’s Forum have stated that women mostly use online fora to complain about treatments and side effects. Posts, they argued, would be overwhelmingly negative in relation to AET, as struggling women seek support and advice. This assumption, however, is partially true. On the one hand, most women did complain about side effects, but on the other, this did not imply a negative attitude towards AET or differentially affected persistence, as our quantitative data on continuation/discontinuation demonstrates.

Finally, users’ posts held in online fora are archived material that allow researchers to retrospectively analyse the perceptions of a range of users, across a large period. This has the merit of facilitating observations and patterns in the data, as guidelines and tests change, as shown in our study. This would be more difficult to identify through interviews, because women who discontinued, for example, would be specific to a particular context, time, and group of participants.

## 5. Conclusions

For early breast cancer patients, extended hormonal therapy beyond 5 years has become a standard treatment. The study identified the challenges that women have faced since guidelines changed in 2013. Our findings revealed the influence of specific factors affecting persistence in an extended treatment context: self-efficacy strategies in managing side effects, as the presence of side effects alone does not mean discontinuation, reassurance about individual risk of recurrence, and quality of life. Recognising women who are ‘undecided’ at some point in their treatment is significant to ensure continuation. These findings show that many women struggle with taking AET for 10 years and may stop taking their medication. Due to the extended duration of treatment, greater attention needs to be made to support patients at prescription of AET and throughout the treatment journey. Interventions such as training of healthcare professionals including risk communication, coping strategies, medication reviews by clinical pharmacists, and re-planning of services in follow-up care, should better support women’s needs in extended hormonal therapy.

## Figures and Tables

**Table 1:** Decision on treatment continuation overall and by treatment scheme, treatment duration, age, and family status.

			Decision on Treatment Continuation						
			Continue		NOT Continue		Undecided		
Category	Subgroup	n	n	%	n	%	n	%	*p*-Value ^3^
Overall sample		130	59	45.4	34	26.2	37	28.5	
By treatment scheme	Prescribed for 10-year treatment	56	35	62.5	8	14.3	13	23.2	
	In 10-year treatment (originally 5-year)	25	14	56.0	5	20.0	6	24.0	
	Planning 10-year treatment (currently in 5-year)	49	10	20.4	21	42.9	18	36.7	<0.001
Treatment duration	2-years or less	36	23	63.9	2	5.6	11	30.6	
	2–5-years	58	16	27.6	24	41.4	18	31.0	
	5–10-years	36	20	55.6	8	22.2	8	22.2	0.001
Age ^1^	<50 years	19	11	57.9	5	26.3	3	15.8	
	50–60 years	37	20	54.1	10	27.0	7	18.9	
	60+ years	20	10	50.0	4	20.0	6	30.0	0.831
Family status ^2^	Lives alone	18	11	61.1	3	16.7	4	22.2	
	Lives with family	48	30	62.5	9	18.8	9	18.7	0.944

^1^ Data available for 76 individuals. ^2^ Data available for 66 individuals. ^3^ *p*-value calculated with Chi-square test.

**Table 2:** Proportion of participants (%) who mentioned various codes, overall and by specific categories.

Codes	Overall Sample (*n* = 130)	By Treatment Scheme				By Decision on Treatment Continuation			
		Prescribed for 10-Year Treatment (*n* = 56)	In 10-Year Treatment (Originally 5-Year) (*n* = 25)	Planning 10-Year Treatment (Currently in 5-Year) (*n* = 49)	*p*-Value ^1^	Continue (*n* = 59)	Not Continue (34)	Undecided (37)	*p*-Value ^1^
	%	%	%	%		%	%	%	
Fear of cancer recurrence	27.7	21.4	40.0	28.6	0.222	39.0	14.7	21.6	0.026
Side effects of medication	92.3	92.9	96.0	89.8	0.625	91.5	91.2	94.6	0.825
Trust in drug’s effectiveness	19.2	16.1	24.0	20.4	0.681	33.2	8.8	8.1	0.003
Family as reason for treatment continuation	5.4	3.6	8.0	6.1	0.687	1.7	5.9	10.8	0.155
Using coping mechanisms to alleviate side effects	22.3	25.0	20.0	20.4	0.813	32.2	14.7	13.5	0.047
Researching the disease using reliable sources	16.9	17.9	4.0	22.5	0.131	15.3	17.7	18.9	0.889
Trust in doctor’s advise	7.8	3.6	12.0	10.2	0.297	5.1	11.8	8.1	0.505
Age as reason for treatment continuation/discontinuation	13.9	16.1	12.0	12.2	0.815	11.9	14.7	16.2	0.823
Quality of life affected by medication	34.6	35.7	36.0	32.7	0.935	18.6	47.1	48.7	0.002
Does not trust medication	5.4	1.8	0.0	12.2	0.025	1.7	11.8	5.4	0.117
Does not trust health professional	9.2	3.6	4.0	18.4	0.020	0.0	23.5	10.8	0.001
Using a test that gives information on risk of cancer recurrence	4.6	5.4	0.0	6.1	0.465	1.7	2.9	10.8	0.101
Other condition that is developed or made worse by taking the drugs	15.4	14.3	20.0	14.3	0.776	13.6	23.5	10.8	0.290
Treatment discontinued by doctor	10.0	1.8	12.0	18.4	0.017	0.0	38.2	0.0	<0.001
Brand change (use of the same drug by different brands)	6.9	8.9	12.0	2.0	0.206	10.2	5.9	2.7	0.360
Switching medication due to intolerance of the first option	19.2	23.2	24.0	12.2	0.290	25.4	5.9	21.6	0.064
Not coping with side effects	25.4	28.6	28.0	20.4	0.597	8.5	41.2	37.8	<0.001

^1^ *p*-values are calculated with Chi-square test.

**Table 3:** Crude and multivariable adjusted association between mentioning of specific codes and decision on treatment continuation (calculated using logistic regression).

Codes	Model	Decision on Continuation						
		Continue	NOT Continue			Undecided		
		OR	OR	(95%CI)	*p*-Value	OR	(95%CI)	*p*-Value
Fear of cancer recurrence	model 1	1 (reference)	0.27	(0.09–0.80)	0.018	0.43	(0.17–1.11)	0.080
	model 2	1 (reference)	0.13	(0.04–0.49)	0.002	0.29	(0.09–0.90)	0.033
Trust in drug’s effectiveness	model 1	1 (reference)	0.20	(0.05–0.75)	0.017	0.19	(0.05–0.68)	0.011
	model 2	1 (reference)	0.13	(0.03–0.61)	0.010	0.12	(0.03–0.55)	0.006
Using coping mechanisms to alleviate side effects	model 1	1 (reference)	0.36	(0.12–1.08)	0.070	0.33	(0.11–0.98)	0.045
	model 2	1 (reference)	0.26	(0.07–0.95)	0.041	0.25	(0.07–0.86)	0.029
Quality of life affected by medication	model 1	1 (reference)	3.88	(1.52–9.93)	0.005	4.13	(1.65–10.36)	0.002
	model 2	1 (reference)	9.05	(2.81–29.1)	<0.001	8.75	(2.83–27.06)	<0.001
Does not trust medication or health professional	model 1	1 (reference)	20.88	(2.50–173.7)	0.005	9.06	(1.01–80.97)	0.049
	model 2	1 (reference)	22.79	(1.89–279.9)	0.014	14.27	(1.06–192.0)	0.045
Not coping with side effects	model 1	1 (reference)	7.56	(2.41–23.70)	0.001	6.57	(2.12–20.39)	0.001
	model 2	1 (reference)	16.47	(4.02–67.58)	<0.001	12.26	(3.17–47.37)	<0.001

Model 1: Unadjusted (crude), Model 2: Adjusted for treatment type, treatment duration (categorical), family status, and age group (for the latter two variables, participants with missing data were included as a separate category). OR—Odds Ratio.

## Data Availability

The data presented in this study are available upon request from the corresponding author. The data are not publicly available due to the fact that the data collected and paraphrased for this study were originally archived on the Internet by a third party (Breast Cancer Now Forum).

## Supplementary Materials

The following are available online at https://www.mdpi.com/article/10.3390/healthcare9060688/s1, Table S1: Proportion of participants who mentioned various codes in the forum, by age group, family status, and treatment duration.
