# Supplementary material for: Extending Adjuvant Endocrine Therapy for 10 Years: A Mixed-Methods Analysis of Women’s Decision Making in an Online Breast Cancer Forum
Source: Healthcare (Basel). 2021 Jun 7;9(6):688. doi: 10.3390/healthcare9060688 (PMC8227818; doi:10.3390/healthcare9060688)
Supplement: Supplementary file 1 [file healthcare-09-00688-s001.zip › healthcare-1238852-supplementary.pdf]

Table S1. Proportion of participants who mentioned various codes in the forum, by age group, family status and treatment duration

Codes	By Age				By family status			By treatment duration			
	<50yr (n = 19)	50-60yr (n = 37)	60+yr (n = 20)	p-value [1]	Lives alone (n = 18)	Lives with family (n = 48)	p-value[1]	<2yr (n = 36)	2-5yr (n = 58)	5-10yr (n = 36)	p-value [1]
	%	%	%		%	%		%	%	%	
Fear of cancer recurrence	15.8	40.5	30.0	0.166	22.2	33.3	0.382	19.4	25.9	38.9	0.167
Side effects of medication	94.7	94.6	95.0	0.998	88.9	95.8	0.292	94.4	91.4	91.7	0.851
Trust in drug`s effectiveness	26.3	18.8	20.0	0.806	22.2	22.9	0.952	19.4	13.8	27.8	0.247
Family as reason for treatment continuation	5.3	5.4	5.0	0.998	5.6	12.5	0.414	5.7	5.2	5.6	0.995
Using coping mechanisms to alleviate side effects	26.3	18.9	15.0	0.664	22.2	22.9	0.952	25.0	24.1	16.7	0.630
Researching the disease using reliable sources	21.1	16.2	5.0	0.332	33.3	6.3	**0.004**	19.4	22.1	5.6	0.095
Trust in doctor`s advise	5.3	10.8	15.0	0.610	0.0	10.4	0.154	2.8	8.6	11.1	0.389
Age as reason for treatment continuation/discontinuation	36.8	16.2	15.0	0.149	16.7	16.7	0.999	22.2	12.1	8.3	0.203
Quality of life affected by medication	42.1	37.8	40.0	0.952	44.4	35.4	0.501	41.7	31.0	33.3	0.564
Does not trust medication	5.3	2.7	5.0	0.862	5.6	2.1	0.464	2.8	10.3	0.0	0.070
Does not trust health professional	10.5	10.8	10.0	0.995	11.1	6.3	0.506	0.0	15.5	8.3	0.040
Using a test that gives information on risk of cancer recurrence	0.0	2.7	15.0	0.069	16.7	2.1	**0.027**	8.3	5.2	0.0	0.233
Other condition that is developed or made worse by taking the drugs	10.5	21.6	10.0	0.397	16.7	14.6	0.833	11.1	15.5	19.4	0.618
Treatment discontinued by doctor	0.0	10.8	10.0	0.336	5.6	6.3	0.916	0.0	15.5	11.1	0.050
Brand change (use of the same drug by different brands)	10.5	8.1	10.0	0.947	5.6	10.4	0.541	8.3	3.5	11.1	0.337

Switching medication due to intolerance of the first option	0.0	29.7	20.0	**0.030**		11.1	29.2	0.127		13.9	19.0	25.0	0.488
Not coping with side effects	31.6	24.3	5.0	0.100		5.6	25.0	0.077		25.0	20.7	33.3	0.391

[1] P-values are calculated with Chi-square test
